# qPMS9: An Efficient Algorithm for Quorum Planted Motif Search

**DOI:** 10.1038/srep07813

**Published:** 2015-01-15

**Authors:** Marius Nicolae, Sanguthevar Rajasekaran

**Affiliations:** 1 Department of Computer Science and Engineering University of Connecticut, Storrs, CT, USA

## Abstract

Discovering patterns in biological sequences is a crucial problem. For example, the identification of patterns in DNA sequences has resulted in the determination of open reading frames, identification of gene promoter elements, intron/exon splicing sites, and SH RNAs, location of RNA degradation signals, identification of alternative splicing sites, etc. In protein sequences, patterns have led to domain identification, location of protease cleavage sites, identification of signal peptides, protein interactions, determination of protein degradation elements, identification of protein trafficking elements, discovery of short functional motifs, etc. In this paper we focus on the identification of an important class of patterns, namely, motifs. We study the (*ℓ*, *d*) motif search problem or Planted Motif Search (PMS). PMS receives as input *n* strings and two integers *ℓ* and *d*. It returns all sequences *M* of length *ℓ* that occur in each input string, where each occurrence differs from *M* in at most *d* positions. Another formulation is quorum PMS (qPMS), where the motif appears in at least *q*% of the strings. We introduce qPMS9, a parallel exact qPMS algorithm that offers significant runtime improvements on DNA and protein datasets. qPMS9 solves the challenging DNA (*ℓ*, *d*)-instances (28, 12) and (30, 13). The source code is available at https://code.google.com/p/qpms9/.

The Planted Motif Search (PMS) problem, also known as the (*l*, *d*)-motif problem, has been introduced in Ref. 1 with the aim of detecting motifs and significant conserved regions in a set of DNA or protein sequences. PMS receives as input *n* biological sequences and two integers *ℓ* and *d*. It returns all possible biological sequences *M* of length *ℓ* such that *M* occurs in each of the input strings, and each occurrence differs from *M* in at most *d* positions. Any such *M* is called a motif.

Buhler and Tompa^2^ have employed PMS algorithms to find known transcriptional regulatory elements upstream of several eukaryotic genes. In particular, they have used orthologous sequences from different organisms upstream of four different genes: preproinsulin, dihydrofolate reductase (DHFR), metallothioneins, and c-fos. These sequences are known to contain binding sites for specific transcription factors. Their algorithm successfully identified the experimentally determined transcription factor binding sites. They have also employed their algorithm to solve the ribosome binding site problem for various prokaryotes. Eskin and Pevzner^3^ used PMS algorithms to find composite regulatory patterns using their PMS algorithm called MITRA. They have employed the upstream regions involved in purine metabolism from three *Pyrococcus* genomes. They have also tested their algorithm on four sets of *S.cerevisiae* genes which are regulated by two transcription factors such that the transcription factor binding sites occur near each other. Price, et al.^4^ have employed their PatternBranching PMS technique to find motifs on a sample containing CRP binding sites in *E.coli*, upstream regions of many organisms of the eukaryotic genes: preproinsulin, DHFR, metallothionein, & c-fos, and a sample of yeast promoter regions.

A problem that is very similar to (*ℓ*, *d*) motif search is the Closest Substring problem. The Closest Substring problem is essentially the PMS problem where the aim is to find the smallest *d* for which there exists at least one motif. These two problems have applications in PCR primer design, genetic probe design, discovering potential drug targets, antisense drug design, finding unbiased consensus of a protein family, creating diagnostic probes and motif finding (see e.g.^5^). Therefore, the development of efficient algorithms for solving the PMS problem constitute an active interest in biology and bioinformatics.

In a practical scenario, instances of the motif may not appear in all of the input strings. This has led to the introduction of a more general formulation of the problem, called quorum PMS (qPMS). In qPMS we are interested in motifs that appear in at least *q* percent of the *n* input strings. Therefore, the PMS problem is the same as qPMS when *q* = 100%.

The Closest Substring problem is NP-Hard^5^. The Closest Substring problem can be solved by a linear number of calls to PMS. Therefore, there is a polynomial time reduction from Closest Substring to PMS, which means that the PMS problem is also NP-Hard. Because of this, all known exact algorithms have an exponential runtime in the worst case. Thus, it is important to develop efficient algorithms in practice. The practical performance of PMS algorithms is typically evaluated on datasets generated as follows (see refs 1, 6): 20 DNA/protein strings of length 600 are generated according to the independent identically distributed (i.i.d.) model. Similarly, a random motif (*ℓ*-mer) *M* is generated and “planted” at a random location in each input string (or in *q*% of the input strings for qPMS). Every planted instance of the motif is mutated in exactly *d* positions.

**Definition 1.** *An (ℓ, d) instance is defined to be a* **challenging instance** *if d is the largest integer for which the expected number of motifs of length ℓ that would occur in the input by random chance does not exceed a constant* (*500 in this paper, same as in* Ref. 7).

Intuitively the more we increase *d*, the more we increase the search space. However, if we increase *d* too much, we find many motifs just by random chance (spurious motifs). According to the above definition, the challenging instances for PMS are (13, 4), (15, 5), (17, 6), (19, 7), (21, 8), (23, 9), (25, 10), (26, 11), (28, 12), (30, 13), etc.

Note that in this paper we only address exact algorithms, which find all the existing motifs. Most of the exact PMS algorithms use a combination of two fundamental techniques. One is a sample driven technique and the other is a pattern driven technique. In the sample driven stage, the algorithm selects a tuple of *ℓ*-mers coming from distinct input strings. In the pattern driven stage, the algorithm generates the common *d*-neighborhood of the *ℓ*-mers in the tuple. Each such *ℓ*-mer becomes a motif candidate. The size of the tuple is usually fixed to a value such as 1 (see e.g.^6^,^8^,^9^), 2 (see e.g.^10^), 3 (see e.g.^11^,^12^,^13^,^14^) or *n* (see e.g.^1^,^15^). In contrast, PMS8^7^ and qPMS9 (this paper) utilize a variable tuple size, which adapts to the problem instance under consideration.

There are many PMS algorithms in the literature. In a previous paper^7^ we have introduced the PMS8 algorithm. In the same paper we have performed a comparison between PMS8 and all the exact algorithms we could find in the literature of the previous five years. We have shown that PMS8 outperforms these algorithms. Ever since the publishing of PMS8, one other exact qPMS algorithm has been published, called TraverStringRef^11^. Therefore, in this paper we compare qPMS9 with PMS8 and TraverStringRef.

The TraverStringRef algorithm^11^ is an algorithm for the qPMS problem, based on the earlier qPMS7^14^ algorithm. qPMS7^14^ can solve, for example, the challenging DNA instance (23,9) whereas TraverStringRef^11^ can solve (25,10), in a reasonable amount of time (no more than two days using commodity processors). In the case of the PMS problem, the PMS8 algorithm^7^ can solve the DNA instances (25,10), on a single core machine, and (26,11) on a multi-core machine. We have used PMS8 as the basis for the new qPMS9 algorithm. The qPMS9 algorithm extends PMS8 in several ways. First, qPMS9 introduces a search procedure which significantly increases performance by allowing for better pruning of the search space. Second, qPMS9 adds support for solving the qPMS problem, which was lacking in PMS8. We compare qPMS9 with PMS8^7^ and TraverStringRef^11^ on several DNA and protein instances.

## Methods

We start by defining the PMS and qPMS problems more formally. A string of length *ℓ* is called an *ℓ*-mer. Given two *ℓ*-mers *u* and *v*, the number of positions where the two *ℓ*-mers differ is called their Hamming distance and is denoted as *Hd*(*u*, *v*). For any string *T*, we denote the substring of *T* starting at position *i* and ending at position *j* by *T*[*i*..*j*].

**Definition 2.** *The PMS problem: Given n sequences s_1_, s_2_, …, s_n_, over an alphabet *Σ*, and two integers ℓ and d, identify all ℓ-mers M, M ∈ *Σ*^l^, such that ∀i, 1 ≤ i ≤ n, ∃j_i_, 1 ≤ j_i_ ≤ |s_i_| − l + 1, such that Hd(M, s_i_*[*j_i_..j_i_ + l − *1]*) ≤ d*.

**Definition 3.** *The qPMS problem: same as the PMS problem, however the motif appears in at least q% of the strings, instead of all of them. PMS is a special case of qPMS for which q* = *100%*.

Another useful notion is that of a *d*-neighborhood. Given a tuple of *ℓ*-mers *T* = (*t*_1_, *t*_2_, …, *t_s_*), the common *d*-neighborhood of *T* includes all the *ℓ*-mers *r* such that *Hd*(*r*, *t_i_*) ≤ *d*, µ1 ≤ *i* ≤ *s*.

We now define the consensus *ℓ*-mer and the consensus total distance for a tuple of *ℓ*-mers. The consensus *ℓ*-mer for a tuple of *ℓ*-mers *T* = (*t*_1_, …, *t_k_*) is an *ℓ*-mer *u* where *u*[*i*] is the most common character among (*t*_1_[*i*], *t*_2_[*i*], …, *t_k_*[*i*]) for each 1 ≤ *i* ≤ *ℓ*. If *p* is the consensus *ℓ*-mer for *T* then the consensus total distance of *T* is defined as 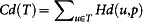. While the consensus string is generally not a motif, the consensus total distance provides a lower bound on the total distance between any motif and a tuple of *ℓ*-mers.

### qPMS9

As indicated previously, most of the motif search algorithms combine a sample driven approach with a pattern driven approach. In the sample driven part, tuples of *ℓ*-mers (*t*_1_, *t*_2_, …, *t_k_*) are generated, where *t_i_* is an *ℓ*-mer in *S_i_*. Then, in the pattern driven part, for each tuple, its common *d*-neighborhood is generated. Every *ℓ*-mer in the neighborhood is a candidate motif. In PMS8^7^ and qPMS9, the tuple size *k* is variable. By default, a good value for *k* is estimated automatically based on the input parameters (see Ref. 7 for details), or *k* can be user specified.

### Tuple Generation

In the sample driven part of PMS8, tuples *T* = (*t*_1_, *t*_2_, …, *t_k_*), where *t_i_* is an *ℓ*-mer from string *s_i_*, ∀*i* = 1..*k*, are generated based on the following principles. First, if *T* has a common *d*-neighborhood, then every subset of *T* has a common *d*-neighborhood. Second, for a motif to exist, there has to be at least one *ℓ*-mer *u* in each of the remaining strings *s_k_*_ + 1_, *s_k_*_ + 2_, …, *s_n_* such that *T* ∪ {*u*} has a common *d*-neighborhood. We call such *ℓ*-mers *u* “alive” with respect to tuple *T*. As we add *ℓ*-mers to *T* we update the alive *ℓ*-mers and reorder the strings in increasing order of the number of alive *ℓ*-mers. This reordering reduces the running time because it leads to generating fewer tuples overall.

**Figure 1:**
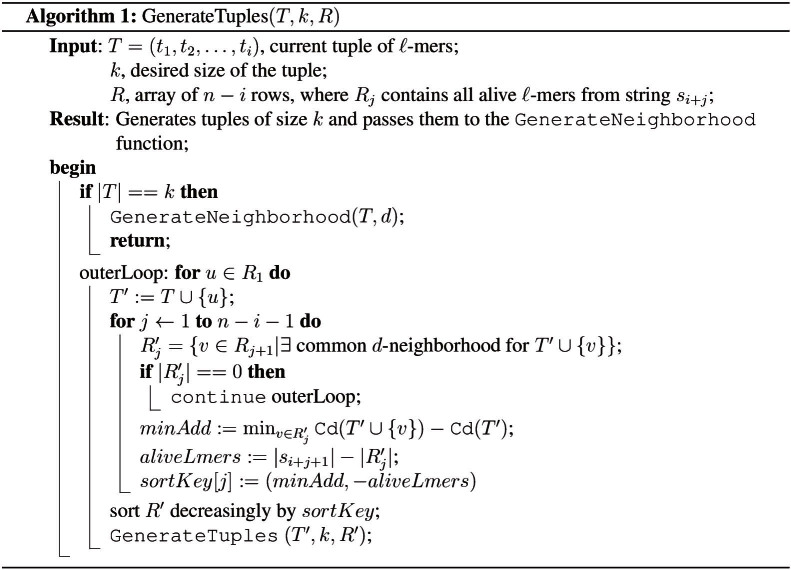
This pseudocode generates tuples of *ℓ*-mers that can potentially have common neighbors, for the PMS problem.

In qPMS9 we change the criteria by which the strings are reordered, as follows. Let *T* be the current tuple of *ℓ*-mers and let *u* be an alive *ℓ*-mer with respect to *T*. If we add *u* to *T*, then the consensus total distance of *T* increases. We compute this additional distance *Cd*(*T*∪{*u*}) − *Cd*(*T*). For each of the remaining strings, we compute the minimum additional distance for any alive *ℓ*-mer in that string. Then we sort the strings decreasingly by the minimum additional distance. Therefore, we give priority to the string with the largest minimum additional distance. If two strings have the same minimum additional distance, we give priority to the string with fewer alive *ℓ*-mers. The intuition is that larger additional distance could indicate more “diversity” among the *ℓ*-mers in the tuple, which means smaller common *d*-neighborhoods. The pseudocode for generating tuples *T* is given in Figure 1. We invoke the algorithm as *GenTuples*({}, *k*, *R*) where the matrix *R* contains all the *ℓ*-mers in all the input strings, grouped as one row per string.

### Neighborhood Generation

For every tuple *T*, obtained as described in the previous section, we generate the common *d*-neighbors of the *ℓ*-mers in the tuple. In qPMS9, the neighbor generation uses the same process as in PMS8^7^. For the sake of completeness, we briefly review the process.

Given a tuple *T* = (*t*_1_, *t*_2_, …, *t_k_*) of *ℓ*-mers, we want to generate all *ℓ*-mers *M* such that *Hd*(*t_i_*, *M*) ≤ *d*, ∀*i* = 1..*k*. We traverse the tree of all possible *ℓ*-mers. A node at depth *r*, which represents an *r*-mer, is not explored deeper if certain pruning conditions are met. Necessary and sufficient conditions for 2 and 3 *ℓ*-mers to have a common neighbor are given in Ref. 7. The same paper gives necessary conditions for more than 3 *ℓ*-mers to have a common neighbor. The interested reader is referred to the PMS8 paper^7^ for a more in depth description of neighborhood generation.

### Adding Quorum Support

**Figure 2:**
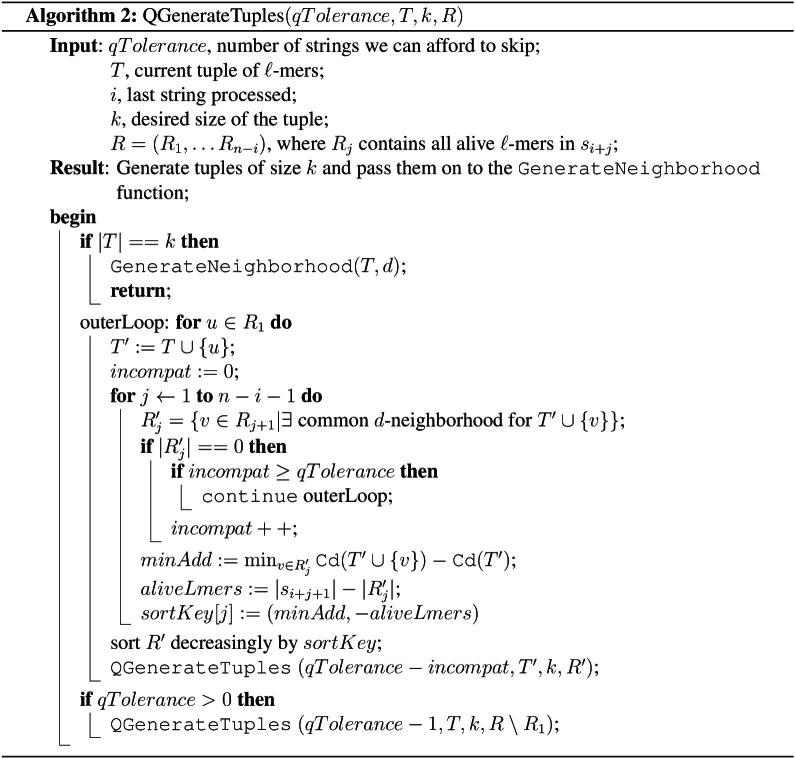
This pseudocode generates tuples of *ℓ*-mers that can potentially have common neighbors, for the qPMS problem.

We extend the algorithm to solve the qPMS problem. In the qPMS problem, when we generate tuples, we may “skip” some of the strings entirely. This translates to the implementation as follows: in the PMS version we successively try every alive *ℓ*-mer in a given string by adding it to the tuple *T* and recursively calling the algorithm for the remaining strings. For the qPMS version we have an additional step where, if the value of *q* permits, we skip the current string and try *ℓ*-mers from the next string. At all times we keep track of how many strings we have skipped. The pseudocode for this algorithm is given in Figure 2. We invoke the algorithm as *QGenerateTuples*(*n* − *Q* + 1, {}, 0, *k*, *R*) where 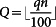 and *R* contains all the *ℓ*-mers in all the strings.

### Parallel Algorithm

In PMS8^7^ the search space is split into *m* = |*s*_1_| − *ℓ* + 1 independent subproblems *P*_1_, *P*_2_, …, *P_m_*, where *P_i_* explores the *d*-neighborhood of *ℓ*-mer *s*_1_[*i*..*i* + *ℓ* − 1]. In the parallel implementation, processor 0 acts as both a master and a worker, the other processors are workers. Each worker requests a subproblem from the master, solves it, then repeats until all subproblems have been solved. Communication between processors is done using the Message Passing Interface (MPI).

In qPMS9, we extend the previous idea to the *q* version. We split the problem into subproblems *P*_1,1_, *P*_1,2_, …, 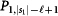, *P*_2,1_, *P*_2,2_, …, 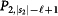, …, *P_r_*_,1_, *P_r_*_,2_, …, 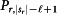 where *r* = *n* − *Q* + 1 and 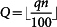. Problem *P_i_*_,*j*_ explores the *d*-neighborhood of the *j*-th *ℓ*-lmer in string *s_i_* and searches for *ℓ*-mers *M* such that there are *Q* − 1 instances of *M* in strings *s_i_*_+1_, …, *s_n_*. Notice that *Q* is fixed, therefore subproblems *P_i_*_,*j*_ get progressively easier as *i* increases.

### Test Data Generation

As mentioned in the introduction, PMS algorithms are typically tested on datasets generated as follows. 20 strings of length 600 each are generated from the i.i.d. We choose an *ℓ*-mer *M* as a motif and plant modified versions of it in *q*% of the *n* strings. Each planted instance is modified in *d* random positions.

It is useful to estimate how many “spurious” motifs (motifs expected by random chance) will be found in a random sample. For that, we make the following observations. The probability that a random *ℓ*-mer *u* is within distance at most *d* from another *ℓ*-mer *v* is


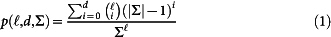


The probability that an *ℓ*-mer is within distance *d* from any of the *ℓ*-mers in a string *S* of length *m* is:


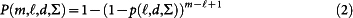


The probability that an *ℓ*-mer is within distance *d* from at least *q* out of *n* strings of length *m* each is:


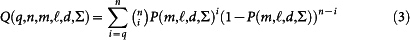


Therefore, the expected number of motifs for a given qPMS instance is: |Σ|*^ℓ^Q*(*q*, *n*, *m*, *ℓ*, Σ). Based on these formulas, we compute for every *ℓ* the largest value of *d* such that the number of spurious motifs does not exceed 500. These values are presented in table 1 for DNA and table 2 for protein.

## Results

In this section we analyze the running times of PMS8^7^, TraverStringRef^11^ and qPMS9, on several synthetic DNA and protein instances. For every instance of the problem we generated 5 datasets as described in the Methods section. For *q* = 100% we compare all three algorithms, for *q* = 50% we compare only the algorithms that solve the quorum PMS problem: TraverStringRef and qPMS9. All programs were executed on the Hornet cluster at the University of Connecticut, which is a highend, 104-node, 1408-core High Performance Computing cluster. For our experiments we used Intel Xeon X5650 Westmere cores. Most results refer to single core execution, unless specified otherwise.

In table 3 we compare the three algorithm on DNA data when *q* = 100%. In table 4 we show a similar comparison on protein data.

In table 5 we compare TraverStringRef and qPMS9 on DNA data when *q* = 50%. In table 6 we compare TraverStringRef and qPMS9 on protein data when *q* = 50%.

**Figure 3:**
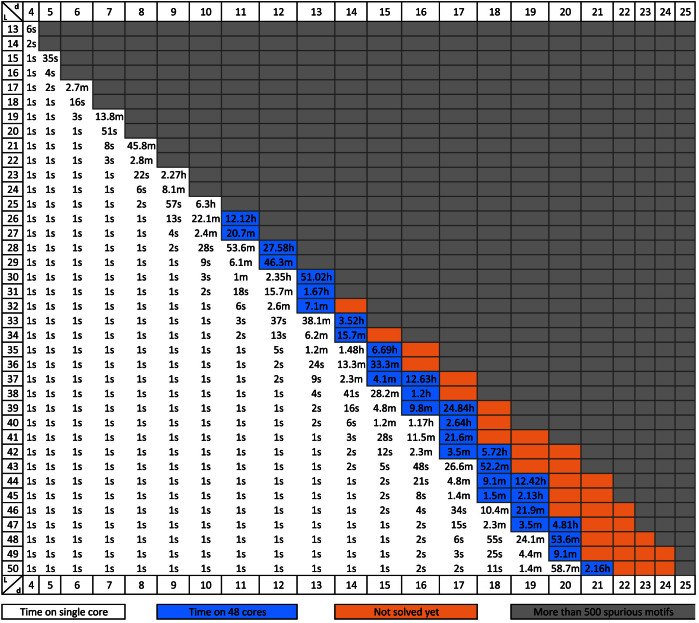
qPMS9 runtimes on DNA datasets for multiple combinations of *ℓ* and *d* where *q* = 100%. The runtimes are averages over 5 random datasets. The times are given in hours (h) minutes (m) or seconds (s). Grey cells indicate instances that are expected to have more than 500 motifs by random chance (spurious motifs). Blue cells indicate that the program used 48 cores whereas white cells indicate single core execution. Instances in orange could not be solved efficiently.

**Figure 4:**
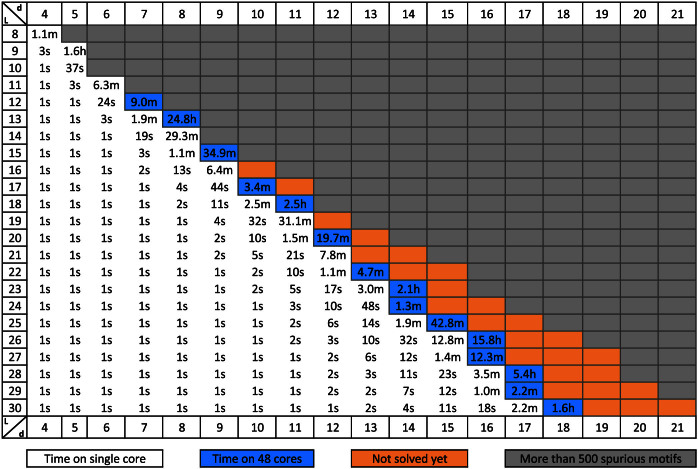
qPMS9 runtimes on protein datasets for multiple combinations of *ℓ* and *d* where *q* = 100%. The runtimes are averages over 5 random datasets. The times are given in hours (h) minutes (m) or seconds (s). Grey cells indicate instances that are expected to have more than 500 motifs by random chance (spurious motifs). Blue cells indicate that the program used 48 cores whereas white cells indicate single core execution. Instances in orange could not be solved efficiently.

In Figure 3 we present the running time of qPMS9 on DNA datasets for all combinations of *ℓ* and *d* with *ℓ* up to 50 and *d* up to 25, with *q* = 100%. In Figure 4 we present the running time of qPMS9 on protein datasets for all combinations of *ℓ* and *d* with *ℓ* up to 30 and *d* up to 21, with *q* = 100%.

## Discussion

We have presented qPMS9, an efficient algorithm for Quorum Planted Motif Search. The algorithm is based on the PMS8 algorithm^7^. qPMS9 includes a new procedure for exploring the search space and adds support for the quorum version of PMS. We compared qPMS9 with two state of the art algorithms and showed that qPMS9 is very competitive. qPMS9 is the first algorithm to solve the challenging DNA instances (28, 12) and (30, 13). qPMS9 can also efficiently solve instances with larger *ℓ* and *d* such as (50, 21) for DNA data or (30, 18) for protein data.

For future work, one of our reviewers kindly pointed out that our approach of filtering *ℓ*-mers for Hamming Distances could benefit for the work in Ref. 16.

## Figures and Tables

**Table 1:** Maximum value of *d* such that the expected number of spurious motifs in random datasets does not exceed 500, for *ℓ* up to 50 and *q* between 50% and 100%, on DNA data

	max d		
L	*q* = 50%	*q* = 75%	*q* = 100%
13	3	3	4
14	3	4	4
15	4	4	5
16	4	5	5
17	4	5	6
18	5	6	6
19	5	6	7
20	6	7	7
21	6	7	8
22	7	8	8
23	7	8	9
24	8	9	9
25	8	9	10
26	9	10	11
27	9	10	11
28	10	11	12
29	10	11	12
30	11	12	13
31	11	12	13
32	12	13	14
33	12	13	14
34	13	14	15
35	13	15	16
36	14	15	16
37	14	16	17
38	15	16	17
39	15	17	18
40	16	17	18
41	16	18	19
42	17	18	20
43	17	19	20
44	18	19	21
45	18	20	21
46	19	21	22
47	19	21	22
48	20	22	23
49	20	22	24
50	21	23	24

**Table 2:** Maximum value of *d* such that the expected number of spurious motifs in random datasets does not exceed 500, for *ℓ* up to 30 and *q* between 50% and 100%, on protein data

	max d		
L	*q* = 50%	*q* = 75%	*q* = 100%
9	4	4	5
10	4	5	5
11	5	6	6
12	6	6	7
13	6	7	8
14	7	8	8
15	8	9	9
16	9	9	10
17	9	10	11
18	10	11	11
19	11	12	12
20	11	12	13
21	12	13	14
22	13	14	15
23	14	15	15
24	14	15	16
25	15	16	17
26	16	17	18
27	16	18	19
28	17	18	19
29	18	19	20
30	19	20	21

**Table 3:** Runtimes for DNA data when *q* = 100%. The time is given in hours (h), minutes (m) or seconds (s), averaged over 5 datasets

(*ℓ*, *d*)	TraverStringRef	PMS8	qPMS9
(13,4)	14 s	7 s	**6 s**
(15,5)	55 s	48 s	**34 s**
(17,6)	3.5 m	5.2 m	**2.7 m**
(19,7)	14.5 m	26.6 m	**13.4 m**
(21,8)	59.8 m	1.64 h	**45.4 m**
(23,9)	4.08 h	5.48 h	**2.26 h**
(25,10)	17.55 h	15.45 h	**6.3 h**

**Table 4:** Runtimes for protein data when *q* = 100%. The time is given in hours (h), minutes (m) or seconds (s), averaged over 5 datasets. TL means that the program runs for more than 24 h

(*ℓ*, *d*)	TraverStringRef	PMS8	qPMS9
(10,5)	2.6 m	42 s	**37 s**
(11,6)	1.67 h	11 m	**6.1 m**
(13,7)	58.2 m	2.6 m	**19 s**
(14,8)	TL	1.03 h	**29.6 m**
(15,8)	28.5 m	1.2 m	**1.1 m**
(17,9)	16.6 m	45 s	**43 s**
(19,10)	5.9 m	**32 s**	**32 s**
(19,11)	TL	1.23 h	**30.1 m**
(22,12)	3.73 h	1.2 m	**1.1 m**
(24,13)	1.84 h	48 s	**47 s**
(26,14)	30.7 m	**31 s**	32 s
(26,15)	TL	1.19 h	**12.5 m**

**Table 5:** Runtimes for DNA data when *q* = 50%. The time is given in hours (h), minutes (m) or seconds (s), averaged over 5 datasets

Instance	TraverStringRef	qPMS9
(20,6)	3 m	**1.5 m**
(22,7)	12.9 m	**6.3 m**
(23,7)	2.6 m	**48 s**
(24,8)	56 m	**26.3 m**
(25,8)	9.9 m	**3.1 m**
(26,9)	4.31 h	**1.55 h**
(27,9)	39.9 m	**10.6 m**
(28,10)	20.86 h	**5.15 h**
(29,10)	2.89 h	**34.5 m**

**Table 6:** Runtimes for protein data when *q* = 50%. The time is given in hours (h), minutes (m) or seconds (s), averaged over 5 datasets. TL means that the program runs for more than 24 h

Instance	TraverStringRef	qPMS9
(9,4)	11.3 m	**3.7 m**
(11,5)	14 m	**4.1 m**
(12,6)	6.22 h	**57.5 m**
(13,6)	17.4 m	**4.9 m**
(14,7)	5.09 h	**41.3 m**
(15,8)	TL	**4.62 h**
(17,9)	TL	**1.79 h**
(18,9)	2.71 h	**33.1 m**
(20,10)	2.33 h	**33.3 m**
(21,11)	TL	**50.9 m**
